# Percutaneous Nephrolithotomy Can Reduce the Incidence of Sepsis Compared with Flexible Ureteroscopy in Treating Solitary Proximal Ureteral Stone Patients with Positive Urine Culture

**DOI:** 10.1155/2021/9927498

**Published:** 2021-04-12

**Authors:** Qi-Dong Xia, Yu-Feng Wang, Chen-Qian Liu, Jin-Zhou Xu, Jian-Xuan Sun, Yang Xun, Cong Li, Jia Hu, Jun-Lin Lu, Shao-Gang Wang

**Affiliations:** Department of Urology, Tongji Hospital, Tongji Medical College, Huazhong University of Science and Technology, Wuhan, China

## Abstract

**Background:**

Sepsis is a potentially lethal complication for both flexible ureteroscopy (fURS) and percutaneous nephrolithotomy (PCNL). This study is aimed at comparing the sepsis rate after fURS and PCNL and the risk factors for sepsis in patients with solitary proximal ureteral stone.

**Methods:**

We reviewed the data of patients with calculi between 10 mm to 20 mm who underwent fURS or PCNL surgery from Tongji Hospital's database. A total of 910 patients were eligible with 412 fURS cases and 498 PCNL cases. We used univariate analysis and multivariate logistic regression analysis to identify the risk factors for sepsis. Subgroup analysis was performed using logistic regression analysis.

**Results:**

In the cohort, 27 (6.6%) and 19 (3.8%) patients developed sepsis after fURS and PCNL, respectively. Multivariate analysis shows that the risk factors for sepsis are fURS (OR = 3.160, *P* = 0.004), serum WBC ≥ 10,000 cells/*μ*L (OR = 3.490, *P* = 0.008), albumin − globulin ratio < 1.2 (OR = 2.192, *P* = 0.029), positive urine culture (OR = 6.145, *P* < 0.001), and prolonged operation time (OR = 1.010, *P* = 0.046). Subgroup analysis was conducted using potential risk factors: stone size, serum WBC, urine culture, and albumin-globulin ratio (AGR). In subgroup of positive urine culture, patients were more likely to develop sepsis after fURS than PCNL.

**Conclusions:**

PCNL may be a better choice than fURS to reduce postoperative sepsis, especially for patients with positive urine culture.

## 1. Introduction

Urolithiasis is one of the most common diseases in the urinary system. It affects patients globally because of its high incidence rate that is 7-13% in North America, 5-9% in Europe, and 1-5% in Asia [1–3]. Surgery is the main treatment for urolithiasis: extracorporeal shock wave lithotripsy, endoscopic surgery, or laparoscopic surgery. Among them, percutaneous nephrolithotomy (PCNL) and flexible ureteroscopy (fURS) are both recommended to remove 10-20 mm ureteral stone [4].

Sepsis is one of the most intractable surgery complications, which leads to a longer length of stay and even lethal sepsis shock in some cases [5]. According to previous studies, postoperative sepsis is the primary complication with an incidence of 0.3-7.4% in fURS and 0.9-5.9% in PCNL [6–8]. It seems that different surgical procedures may lead to different incidence rate of sepsis. Thus, urologists make efforts to discover risk factors or preoperative predicting factors for postoperative sepsis. There have been several preoperative features identified as risk factors such as positive urine culture, female sex, and diabetes [9]. However, though the preoperative risk factor has been identified, it is hardly helpful for clinicians to make a clinical decision about which surgical procedure to choose, PCNL or fURS. Thus, we aim to compare the occurrence of postoperative sepsis between fURS and PCNL and analyze the difference of risk factors.

Besides, PCNL is usually utilized to treat larger stone than fURS [10, 11]. Considering the heterogeneity of the stone size between patients may influence the sepsis incidence, we conducted a retrospective clinical data collection of patients with solitary proximal ureteral stone with stone size between 10 and 20 mm. We performed univariate and multivariate analyses to discover the risk factors for postoperative sepsis in our cohort. Our works may provide evidence for clinicians to make surgical choice.

## 2. Materials and Methods

The research was approved by the Ethics Committee of Tongji Medical College (2019S1035). The retrospective study included patients from January 2012 to December 2018. The inclusion criteria were (1) unilateral, solitary, and proximal ureteral stones; (2) PCNL or fURS to treat urolithiasis; (3) stone size ranging from 10 mm to 20 mm; (4) patient age ≥ 18 years. The exclusion criteria were anatomical abnormality: solitary kidney, horseshoe kidney, transplant kidney, and kidney duplication.

The primary outcome was postoperative sepsis. According to the 2001 International Sepsis Definitions Conference, postoperative sepsis was defined as the concurrence of infection and at least two of the following criteria with 48 hours of surgery: (1) heart rate > 90/minute, (2) respiratory rate > 20/minute, (3) body temperature > 38°C, and leukocyte count < 4,000 cells/*μ*L or >12,000 cells/*μ*L.

The patient data was retrospectively collected from the hospital's database. Preoperative factors were recorded such as patient age, sex, body mass index (BMI), comorbidities (diabetes, coronary heart disease, paraplegia, and hypertension), stone size and laterality, presence of hydronephrosis and indwelling stent, hematological tests (serum white blood cell [WBC], neutrophil, and lymphocyte), biochemical tests (creatinine, cholesterol, albumin, and globulin), urine tests (urine WBC and urine nitrite), urine culture, and American Society of Anesthesiologists (ASA) score. Size of ureteral access sheath and flexible ureteroscope for fURS and size of sheath and nephroscope for PCNL were also recorded. Operation time was documented from the commencement of operation to the end of anesthesia. The laboratory tests were routinely performed and obtained for all patients. Patients who have infectious indicators (fever, high serum WBC proportion, or positive urine culture) received at least a full antibiotic regimen for seven days until the tests turned negative. Otherwise, one dose of antibiotics was applied for prophylactic purpose.

Statistical analysis was conducted using Statistical Product and Service Solutions (SPSS) version 24.0. The Student's *t*-test was used to compare continuous variables (expressed by mean ± standard deviation) with a normal distribution. Continuous variables with a skewed distribution were showed as median (interquartile range [IQR]) and compared by the Mann-Whitney *U* test. The chi-square test or Fisher's exact test was utilized to detect the difference between groups with categorical variables (expressed by proportions). The logistic regression method was used to identify the risk factors of sepsis. The difference was considered statistically significant when *P* value <0.05.

## 3. Results

**Figure 1:**
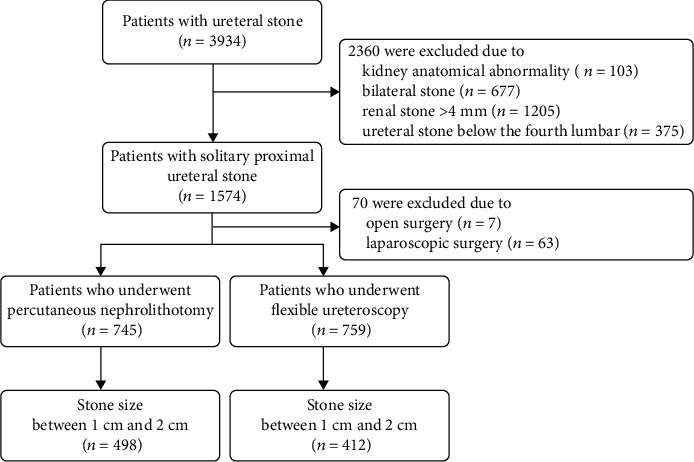
The screening flow chart.

After reviewing 3934 patients with ureteral stone, 2360 were excluded primarily due to kidney anatomical abnormality (*n* = 103), bilateral stone (*n* = 677), renal stone > 4 mm (*n* = 1205), and ureteral stone below the fourth lumbar (*n* = 375). Finally, a total of 910 patients with 10-20 mm solitary proximal ureteral stone were eligible for analysis (Figure 1). Among them, 412 patients underwent fURS, whereas 498 patients received PCNL.

The detailed basic information of the eligible patients is shown in Table 1. Patients who received PCNL had higher rate of hydronephrosis, larger stone size, and longer operation time. The rough sepsis rate of fURS (6.6%) is higher than that of PCNL (3.8%). But the difference is not significant (*P* = 0.061). The multivariate analysis indicates that five variables are independent risk factors of sepsis (Table 2): fURS (OR = 3.160, *P* = 0.004), serum WBC ≥ 10,000 cells/*μ*L (OR = 3.490, *P* = 0.008), albumin − globulin ratio < 1.2 (OR = 2.192, *P* = 0.029), positive urine culture (OR = 6.145, *P* < 0.001), and prolonged operation time (OR = 1.010, *P* = 0.046). Sex (*P* = 0.354) and stone size (*P* = 0.716) are not considered as independent risk factors.

**Figure 2:**
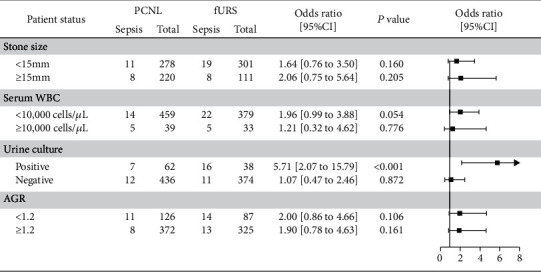
Subgroup analysis to compare sepsis rate between PCNL and fURS.

Urologists are more likely to choose PCNL to treat larger stones. We divided patients into a larger stone size group (15-20 mm) and a smaller stone size group (10-15 mm) to evaluate the sepsis rate between fURS and PCNL (Figure 2). Both groups are not of statistical difference: *P* = 0.160 for smaller stone size group and *P* = 0.205 for larger stone size group. Subgroup analysis using sepsis risk factors is also performed: serum WBC, urine culture, and albumin-globulin ratio (AGR). In the positive urine culture group, patients who receive fURS have 5.71 times the risk of developing sepsis than patients who receive PCNL (*P* < 0.001).

## 4. Discussion

Sepsis is one of the most severe complications in patients who underwent lithotomy, which may both occur after PCNL or fURS [12, 13]. It has been reported that the occurrence rate of sepsis post-fURS or post-PCNL was different. According to previous research, the sepsis rate after fURS reaches 0.3%-7.4% [6, 7], and the sepsis rate after PCNL reaches 0.9%-5.9% [8]. In our study, sepsis after PCNL was 3.8% (19/498) and 6.6% (27/412) after fURS, which shows a consistency with previous studies. Based on the multivariate logistic analysis, we find that surgical option, positive urine culture, serum WBC, and operation time are independent predictors of postoperative sepsis. Patients with positive urine culture are more likely to suffer sepsis after fUSR than PCNL. However, no significant difference in sepsis rate is indicated between PCNL and fURS when urine culture is negative.

We previously report that positive urine culture is an independent predictor for post-fURS sepsis and assemble a nomogram to predict the occurrence of post-fURS sepsis [14]. Uchinda [15] et al. and Blackmur et al. [7] explored the role of bladder urine culture in infectious complications that it is an independent risk factor increasing 3.53 to 4.88 times the risk of infectious complications. It may be because the high intrarenal pressure during the fURS promotes local pathogens and toxins into blood circulation. AGR usually plays a role as a predictor of cancer progression or cancer-specific survival because it reflects patients' nutrition, inflammation, and immunity [16]. We also find it as a predictor of sepsis after endourological stone surgery [14, 17]. In this study, both low AGR and high AGR groups, PCNL, and fURS have similar sepsis rates. From the aspect of reducing operative sepsis, the level of AGR may have little influence on surgical option.

Kreydin and Eisner have systemically summarized the risk factors for sepsis after PCNL [18], and the pre-PCNL factors included positive urine culture, female, nephrostomy, urinary diversion, stone size, hydronephrosis, diabetes, and complicated calculi. Meanwhile, the local urinary system condition is considered the most critical factor related to infectious complications. Besides, more novel predictors for post-PCNL sepsis are identified by researchers. For example, C-reactive protein, albumin, and procalcitonin are considered predictors reflecting the systematic condition of patients [8, 19].

Positive urine culture is a predictor of postoperative sepsis for both fURS and PCNL. The evidence reveals that urine culture can be an essential reference factor for surgical choice to reduce the incidence of sepsis. In patients with 10-20 mm ureteral stone, we find that PCNL is better than fURS when patients have a positive urine culture. Our works can optimize the surgery strategy for patients with a high risk of infection [20, 21].

The main limitation of the study is the single-center retrospective nature, which may cause selection bias. We include a relatively large number of patients to stabilize the results. Subgroup analysis is also performed to compare sepsis incidence in patients with different conditions. Further prospective and multicenter studies are needed.

## 5. Conclusions

In summary, we find that PCNL might be a better choice than fURS to reduce postoperative sepsis, especially when patients have a positive urine culture.

## Figures and Tables

**Table 1:** Basic characteristics of including patients.

Variables	All patients (*n* = 910)	fURS (*n* = 412)	PCNL (*n* = 498)	*P* value
Age (years)	50.0 ± 12.4	49.1 ± 13.2	50.6 ± 11.6	0.074
Male, *n* (%)	588 (64.6)	271 (65.8)	317 (63.7)	0.505
BMI (kg/m^2^)	24.0 ± 3.1	24.2 ± 3.3	23.9 ± 3.0	0.163

*Preoperative urological condition, *n* (%)*				
Hydronephrosis	131 (14.4)	34 (8.3)	97 (19.5)	<0.001
Indwelling stent	63 (6.9)	29 (7.0)	34 (6.8)	0.900

*Stone characteristics*				
Stone size (mm)	13.3 ± 3.2	12.4 ± 2.8	14.1 ± 3.2	<0.001
Left side, *n* (%)	476 (52.3)	208 (50.5)	268 (53.8)	0.317

*Comorbidities, *n* (%)*				
Diabetes	74 (8.1)	39 (9.5)	35 (7.0)	0.180
Coronary heart disease	15 (1.6)	6 (1.5)	9 (1.8)	0.679
Hypertension	210 (23.1)	90 (21.8)	120 (24.1)	0.422

*Urine test*				
Urine WBC, median (IQR) (cells/hpf)	52.0 (19.6-163.7)	46.0 (17.1-127.4)	59.0 (21.3-193.4)	0.024
Positive urine nitrite, *n* (%)	55 (6.0)	23 (5.6)	32 (6.4)	0.595
Positive urine culture, *n* (%)	100 (11.0)	38 (9.2)	62 (12.4)	0.121

*Hematological test (10^9^cells/L)*				
Serum WBC	6.8 ± 2.8	6.9 ± 3.2	6.6 ± 2.5	0.164
Neutrophil	4.1 ± 2.7	4.3 ± 3.0	4.0 ± 2.4	0.169
Lymphocyte	1.9 ± 0.6	1.9 ± 0.6	1.9 ± 0.6	0.943

*Biochemical test*				
Albumin (g/L)	39.5 ± 4.2	39.8 ± 4.3	39.3 ± 4.3	0.662
Globulin (g/L)	29.2 ± 4.6	29.2 ± 4.4	29.2 ± 4.8	0.061
AGR	1.39 ± 0.28	1.39 ± 0.27	1.39 ± 0.29	0.051
Creatinine, median (IQR) (*μ*moI/L)	85.0 (69.0-108.0)	83.0 (68.0-108.0)	86.0 (70.0-109.0)	0.680
Cholesterol (mmol/L)	4.1 ± 0.9	4.1 ± 0.9	4.1 ± 0.9	0.848

*ASA, *n* (%)*				0.150
I	378 (41.5)	157 (38.1)	221 (44.4)	
II	510 (56.0)	243 (59.0)	267 (53.6)	
III	21 (2.3)	12 (2.9)	9 (1.8)	
IV	1 (0.1)	0 (0.0)	1 (0.2)	
Operation time, median (IQR) (min)	85.0 (66.0-110.0)	72.5 (58.0-89.0)	98.0 (78.0-120.0)	<0.001
Postoperative sepsis, *n* (%)	46 (5.1)	27 (6.6)	19 (3.8)	0.061

**Table 2:** Multivariate analysis for the risk factors of sepsis.

Variables	B	OR	95% CI	*P* value
Surgery (ref. PCNL)	1.151	3.160	(1.459 to 6.842)	0.004
Sex (ref. male)	0.324	1.383	(0.697 to 2.747)	0.354
Age (years)	0.019	1.019	(0.990 to 1.048)	0.200
BMI (kg/m^2^)	0.013	1.014	(0.910 to 1.129)	0.807
Stone size (mm)	0.020	1.021	(0.915 to 1.139)	0.716
Indwelling stent	-0.038	0.962	(0.338 to 2.739)	0.943
Diabetes	-0.431	0.650	(0.181 to 2.334)	0.509
Hydronephrosis	-0.519	0.595	(0.168 to 2.107)	0.421
Serum WBC ≥ 10,000 cells/*μ*L	1.250	3.490	(1.391 to 8.758)	0.008
AGR < 1.2	0.785	2.192	(1.082 to 4.442)	0.029
Positive urine culture	1.816	6.145	(2.541 to 14.859)	<0.001
Urine WBC ≥ 50 cells/hpf	0.185	1.203	(0.531 to 2.726)	0.659
Positive urine nitrite	0.676	1.967	(0.750 to 5.157)	0.169
Operation time (min)	0.010	1.010	(1.000 to 1.020)	0.046

Abbreviations: PCNL: percutaneous nephrolithotomy; BMI: body mass index; WBC: white blood cell; AGR: albumin globulin ratio.

## Data Availability

All data that supports the findings of this study is available from the corresponding author upon reasonable request.

## Abbreviations

fURS: Flexible ureteroscopy
PCNL: Percutaneous nephrolithotomy
BMI: Body mass index
WBC: White blood cell
AGR: Albumin-globulin ratio
ASA: American Society of Anesthesiologists
SPSS: Statistical Product and Service Solutions
IQR: Interquartile range.
